# Heat shock protein 90 inhibition abrogates TLR4-mediated NF-κB activity and reduces renal ischemia-reperfusion injury

**DOI:** 10.1038/srep12958

**Published:** 2015-08-07

**Authors:** Stephen O’Neill, Duncan Humphries, George Tse, Lorna P. Marson, Kevin Dhaliwal, Jeremy Hughes, James A. Ross, Stephen J Wigmore, Ewen M. Harrison

**Affiliations:** 1 MRC Centre for Inflammation Research, University of Edinburgh, Edinburgh, EH16 4SA; 2 MRC Centre for Regenerative Medicine, University of Edinburgh, Royal Infirmary of Edinburgh, 49 Little France Crescent, Edinburgh EH16 4SA.

## Abstract

Renal ischemia-reperfusion injury (IRI) is a common cause of acute kidney injury. Toll-like receptor 4 (TLR4) mediates sterile inflammation following renal IRI. Heat shock protein 90 (Hsp90) inhibition is a potential strategy to reduce IRI, and AT13387 is a novel Hsp90 inhibitor with low toxicity. This study assessed if pre-treatment with AT13387 could reduce renal IRI and established if the mechanism of protection involved a reduction in inflammatory signalling. Mice were pre-treated with AT13387 prior to renal IRI. 24 h later, renal function was determined by serum creatinine, kidney damage by tubular necrosis score, renal TLR4 expression by PCR and inflammation by cytokine array. *In vitro*, human embryonic kidney cells were co-transfected to express TLR4 and a secreted alkaline phosphatase NF-κB reporter. Cells were pre-treated with AT13387 and exposed to endotoxin-free hyaluronan to stimulate sterile TLR4-specific NF-κB inflammatory activation. Following renal IRI, AT13387 significantly reduced serum creatinine, tubular necrosis, TLR4 expression and NF-κB-dependent chemokines. *In vitro*, AT13387-treatment resulted in breakdown of IκB kinase, which abolished TLR4-mediated NF-κB activation by hyaluronan. AT13387 is a new agent with translational potential that reduces renal IRI. The mechanism of protection may involve breakdown of IκB kinase and repression of TLR4-mediated NF-κB inflammatory activity.

The rising incidence of acute kidney injury represents a significant global health concern^1^. Renal ischemia-reperfusion injury (IRI) is a common cause of acute kidney injury following blood loss, trauma and surgery^2^. Preventative treatments are currently lacking and apart from supportive renal replacement therapy, no other therapies exist for patients who develop acute ischemic kidney injury. The best treatments presently available are avoidance of further kidney damage through careful resuscitation, effective treatment of sepsis and avoidance of nephrotoxic medications^3^.

It has previously been demonstrated that functional and morphological protection from renal IRI in mice follows pre-treatment with the Heat shock protein (Hsp) 90 inhibitor 17-dimethylamino-ethylamino-17-demethoxygeldanamycin (17-DMAG)^4^. However, the molecular mechanisms of protection from renal IRI offered by Hsp90 inhibitors remain to be fully delineated. Defining these mechanisms are essential for the further development of these agents and translation to patients.

It is now recognised that inflammation is perhaps the most critical pathophysiological process involved in the propagation of renal IRI^2^. Toll-like receptor 4 (TLR4) is a key regulator of the pro-inflammatory transcription factor NF-κB and plays a dominant role in mediating sterile kidney damage following renal IRI^5^. IκB kinase (IKK) activation leads to the dissociation of NF-κB from IκB and its subsequent activation^6^. Hsp90 may be needed to stabilise the IKK^7^. Consequently, Hsp90 inhibitors could cause dissociation of the IKK complex, prevention of NF-κB activation and a reduction in inflammation.

This study investigates the mechanism of protection offered by Hsp90 inhibitors in renal IRI. Specifically, it addresses the hypothesis that Hsp90 inhibition represses TLR4-mediated NF-κB inflammatory activity through breakdown of IKK. AT13387 (Astex Pharmaceuticals) is a new small molecule Hsp90 inhibitor with a low toxicity profile and better translational potential than 17-DMAG. Therefore this study also aims to determine the efficacy of AT13387 in reducing renal IRI^8^.

## Methods

### *In vivo* experiments

#### Animals

Male FVB/n mice aged 6–8 weeks and weighing 20–25 g, were used from in-house colonies. Mice were allowed free access to standard chow and water. The mice were kept in a 12:12-h light-dark cycle. All work involving animals was conducted in accordance with the provisions of the UK Animals (Scientific Procedures) Act 1986. The United Kingdom Home Office approved all experimental protocols. Procedures were performed under Home Office project license 60/3737 and personal license 60/3999.

#### Drugs

The Hsp90 inhibitor AT13387 was kindly provided by Astex Pharmaceuticals^9^. For *in vivo* experiments AT13387 (80 mg/kg free-base equivalent) was dissolved in (2-Hydroxypropyl)-β-cyclodextrin (2HβC) (Sigma-Aldridge, Dorset, UK), which was prepared in distilled water at 17.5% weight per volume. Mice were treated by intra-peritoneal injection 24 h prior to surgery. A 2HβC vehicle-treated group of mice served as the controls.

#### Renal IRI model

An established model of warm IRI of the left kidney together with a right nephrectomy was used^10^. The model was designed to inflict a moderate to severe acute tubular injury with zero animal mortality. Mice were anesthetised with an intra-peritoneal injection of Ketamine (75 mg/kg, Vetalar; Pfizer, Kent, UK) and Medetomidine (1 mg/kg, Domitor, Pfizer). A heated mat maintained body temperature with homeostatic control via a rectal temperature probe (Harvard Apparatus, Boston, MA). A mid-line laparotomy was performed for access to both kidneys through a single incision. The right ureter and renal pedicle were ligated then divided. The right kidney was then removed as a control and placed in Methacarn (70% methanol, 20% chloroform, 10% acetic acid). The pedicle of the left kidney was dissected and occluded using an atraumatic vascular clamp (Micro Serrefine, Fine Science Tools, Linton, UK) for 20 minutes. Following removal of the left pedicle clamp, reperfusion was confirmed visually before closure of the abdomen. The anaesthetic was reversed with Atipamezole (1 mg/kg, Antisedan, Pfizer), and a subcutaneous injection of 0.9% saline (25 ml/kg) and Buprenorphine (0.1 mg/kg) was administered. Animals were recovered in an incubator at 29 °C until tissue collection 24 h later.

#### Tissue collection

Under terminal general anaesthesia, blood was recovered by intra-cardiac puncture, and the serum was stored at −20 °C. Kidneys were placed immediately into Methacarn, RNAlater (Life Technologies, Paisley, UK) or were snap frozen.

#### Scoring of morphological kidney injury

Hematoxylin and eosin-stained sections were evaluated for tubulointerstitial injury. A series of non-overlapping fields (x200) were examined by an observer blinded to the sample number. A scoring system based on the proportion of tubules with necrotic/detached cells was used (tubular necrosis score: 0, none; 1, <30%; 2, 30 –70%; 3, >70%)^4^.

#### Serum creatinine determination

Plasma samples were prepared from whole blood. Creatinine was determined using the creatininase/creatinase specific enzymatic method, utilising a commercial kit (Alpha Laboratories Ltd, Eastleigh, UK) adapted for use on a Cobas Fara centrifugal analyser (Roche Diagnostics Ltd, Welwyn Garden City, UK)^11^.

#### Cytokine array panel

Kidney protein lysates (200 μg per sample) were mixed with biotinylated detection antibodies. The sample/antibody mixture was then incubated with a mouse cytokine array panel (R&D Systems, Abingdon, UK). The panel consisted of a nitrocellulose membrane with capture antibodies for 40 murine cytokines spotted on its surface in a duplicate manner. Cognate immobilized capture antibodies on the membrane bound any cytokine/detection antibody complexes present. Following a wash to remove unbound material, Streptavidin-HRP and chemiluminescent detection reagents were added sequentially followed by development using autoradiography. Light produced at each spot was used to determine the amount of cytokine bound. Mean gray values were quantified using Image J (National Institutes of Health, USA)^12^.

#### Real-time reverse transcriptase–PCR

For the detection of renal TLR4 expression, RNA was extracted from kidneys and purified by TRIzol (Invitrogen, Life Technologies, Paisley, UK). RNA concentration and quality was determined using a Nanodrop spectrophotometer and Agilent Bioanalyzer. Across all samples the mean 260/280 ratio was 2.06 +/− 0.01, and mean RNA integrity number was 7.9 +/− 0.6. cDNA was synthesized using a RT^2^ First Strand Kit (Qiagen, Manchester, UK). Real-time PCR was then performed using a RT^2^ qPCR primer assay for mouse TLR4 (Qiagen, Manchester, UK). Glyceraldehyde 3-phosphate dehydrogenase (GAPDH) was used as the reference gene, and was also quantified using a RT^2^ qPCR primer assay (Qiagen, Manchester UK).

#### Power calculation

For animal experiments in the renal IRI model, power calculations were performed and the number of mice was kept to a minimum required to answer the research question. Creatinine was selected as the primary outcome measure since it is objectively measured and less affected by hydration status than blood urea nitrogen. Although creatinine levels are affected by muscle mass and thus differ according to mouse gender and age, all experiments were performed on male mice of the same age. Previous data was used to the estimate effect size for the potential reduction in serum creatinine. Based on the reduction in creatinine in animals treated with Hsp90 inhibitors that underwent contralateral nephrectomy and 30 minutes renal IRI, an effect size of 1.70 was calculated^4^. With α set at 0.05 and β set at 0.1 this translated to a required sample size of 9 animals per treatment group^13^.

### *In vitro* experiments

#### Cell culture

Cell lines included human embryonic kidney cells (HEK293) (European Collection of Cell Cultures, Porton Down, UK), stably transfected HEK293 expressing TLR4 (HEK293-TLR4) and stably co-transfected HEK293 expressing both TLR4 and a secreted alkaline phosphatase (SEAP) reporter under the transcriptional control of NF-κB (HEK293-TLR4-NF-κB) (Imgenex, San Diego, USA). Cells were maintained in Dulbecco’s modified eagle’s medium (Gibco, Paisley, UK) supplemented with 10% foetal bovine serum, penicillin (50 U/ml), streptomycin (50 μg/ml) and non-essential amino acids (5%). The selection agent for HEK293-TLR4 cells was 10 μg/ml blasticidin (Invivogen, San Diego, CA) and for HEK293-TLR4-NF-κB cells were 10 μg/ml blasticidin, 2 μg/ml puromycin, 200 μg/ml zeocin and 500 μg/ml G418/geneticin (Invitrogen, Carlsbad, CA).

#### Drugs and reagents

17-DMAG was purchased from InvivoGen (San Diego, CA). For *in vitro* experiments, stock solutions were formed for 17-DMAG and AT13387 in Dimethyl sulfoxide (DMSO) (Sigma-Aldridge, Dorset, UK) and further diluted prior to use. DMSO vehicle treated cells served as the control. Pre-treatment with Hsp90 inhibitors was performed in a time window 6 or 12 h prior to ligand stimulation.

#### Reagents

TLR4 grade endotoxin-free hyaluronan was purchased from Enzo Life Sciences (Exeter, UK) and polymyxin B from SERVA (Heidelberg, Germany).

#### Transfection

Transient NF-κB transfections were performed using Fugene HP (Roche, Lewes, East Sussex, UK) in the experiment assessing NF-κB activation in HEK293 cells and HEK293-TLR4 cells, as these cells did not have a stably transfected NF-κB reporter. Transfection efficiency of the NF-κB reporter construct (GL4.32 [luc2P/NF-κB-RE/Hygro]) was controlled by co-transfecting with a control vector (pGL4.73 control vector [hRluc/SV40]) (Promega, Southampton, UK).

#### Western blot

Western blotting was performed as previously described^4^. Briefly, whole cells extracts were produced and protein concentration determined (BioRad, Hemel Hempstead, UK). Proteins were separated by SDS-PAGE (10% Tris/HCl gels) and transferred to nitrocellulose (BioRad, Hemel Hempstead, UK). Nitrocellulose membranes were soaked in blocking buffer then primary antibody. Primary antibodies were from the IKK isoform antibody kit (Cell Signaling Technology, Boston, MA). After washing, the membranes were exposed to a horseradish peroxidase-conjugated secondary antibody. Enhanced chemiluminescence reagent (Amersham, Chalfont St Giles, UK) was applied followed by development with autoradiography. Equality of loading was confirmed by probing for β-actin (BD Biosciences, San Diego, CA). Autoradiography films were uploaded to a Gel Doc (Bio-Rad, Hemel, UK) using Quantity One software before being cropped. The original autoradiography is available in the Supplementary Material.

#### Flow cytometry

Cells were trypsinised, collected and incubated at room temperature with 2% rabbit serum. Further incubation was then performed with either PBS/0.5% Bovine Serum Albumin (BSA)/0.1% azide (unstained control), mouse IgG1 isotype control antibody (Serotec, Oxford, UK) or purified mouse anti-human TLR4 antibody (BD Pharmingen, San Diego, CA) for test samples (0.5 mg/ml at 1:50 dilution or 0.5 μg per test sample). Cells were washed then incubated with either PBS/0.5% BSA/0.1% azide for unstained controls or fluorescein isothiocyanate (FITC)-conjugated goat anti-mouse IgG (Silenus Laboratories, Victoria, Australia) for IgG1 control samples and TLR4-test samples. Cells were washed again and results acquired on a BD Accuri C6 flow cytometer (BD Biosciences, San Jose, USA).

#### Luciferase assay

Luciferase assays were used to investigate NF-κB activity following transient NF-κB transfections in HEK293 and HEK293-TLR4 cells. At 24 h after transfection and following experimentation, cells were processed for luciferase activity using the Dual-Glo luciferase assay system and Turner Biosystems Modulus Microplate analyser (Promega, Southampton, UK). Firefly luciferase luminescence activity was normalised to that of the co-expressed renilla luciferase to determine the fold induction and indicate NF-κB activity. Maximal signal intensity was achieved 3 h following ligand stimulation with hyaluronan.

#### SEAP assa**y**

SEAP assays were used to investigate NF-κB activity in HEK293-TLR4-NF-κB cells as these cells were stably transfected with a SEAP reporter under the transcriptional control of NF-κB. SEAP assays were performed using the SEAPorter assay kit (Imgenex, San Diego, USA). The concentration of SEAP secreted into cell culture supernatant was calculated from a SEAP standard curve and was used to indicate NF-κB activation. The assay was performed 24 h after the addition of ligands as per the manufacturer’s instructions in order to allow adequate time for SEAP to be secreted from cells into the culture medium.

### Statistical analysis

Data are presented as mean and standard error of the mean or in standard boxplots with individual results jittered. Statistical comparisons for parametric continuous data were made using student’s t-test, one-way analysis of variance (ANOVA) and two-way ANOVA without interaction (using the Tukey’s HSD post hoc correction for multiple comparisons). Statistical comparisons for non-parametric data were made using the Mann-Whitney U test. All comparisons were performed in R v3.0.1 (R Foundation for Statistical Computing).

## Results

### AT13387 reduces renal IRI and inflammatory signalling in the kidney

#### AT13387 pre-treatment reduces functional and morphological kidney injury from renal IRI

**Figure 1:**
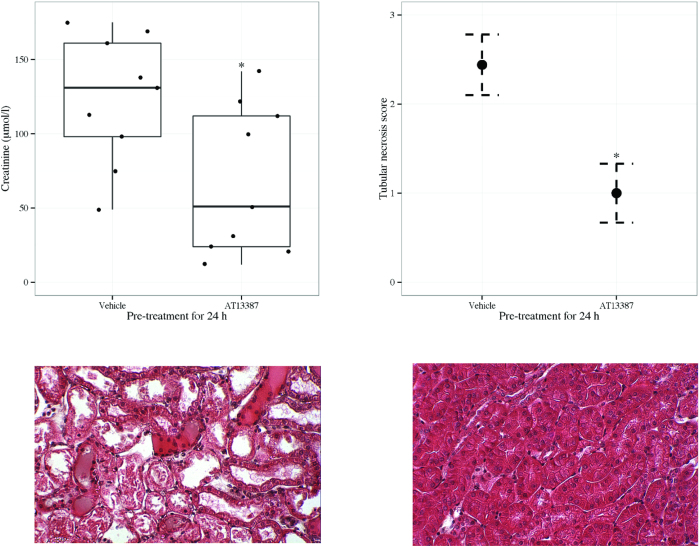
(**a**) Serum creatinine following AT13387 or vehicle pre-treatment and 24 h following renal IRI in FVB/n mice (upper left panel). FVB/n mice were pre-treated with AT13387 or 2HβC vehicle (n = 9 per group). 24 h later, mice were anaesthetised and underwent right nephrectomy and 20 min of left renal pedicle clamping. Following 24 h of recovery, blood was obtained by intra-cardiac puncture and serum creatinine was determined. Results are presented in a standard boxplot with individual results jittered. *p < 0.05 vs. 2HβC vehicle, t-test. (**b**) Tubular necrosis score following AT13387 or vehicle pre-treatment and 24 h following renal IRI in FVB/n mice (upper right panel). The left kidney was harvested and placed in methacarn. Sections were later prepared and stained with haematoxylin and eosin. A blinded observer then determined the tubular necrosis score. Results are presented as mean and standard error of the mean. *p < 0.05 vs. 2HβC vehicle, Mann-Whitney U test. (**c**) Morphological kidney injury following pre-treatment with vehicle and 24 h following renal IRI in FVB/n mice (lower left panel). A representative section of kidney cortex at x 200 magnification is shown. (**d**) Morphological kidney injury following pre-treatment with AT13387 and 24 h following renal IRI in FVB/n mice (lower right panel). A representative section of kidney cortex at x 200 magnification is shown.

On serum analysis, in comparison to vehicle, AT13387 pre-treatment in FVB/n mice significantly reduced creatinine 24 h following renal IRI (AT13387 vs. 2HβC vehicle, p < 0.05, t-test) (Fig. 1a). AT13387 pre-treatment in FVB/n mice also reduced tubular necrosis score (AT13387 vs. 2HβC vehicle, p < 0.05, Mann-Whitney U test) (Fig. 1b) on histological assessment 24 h following renal IRI (Fig. 1c,d).

#### AT13387 pre-treatment reduces the expression of TLR4 and inflammatory chemokines in the kidney following renal IRI

**Figure 2:**
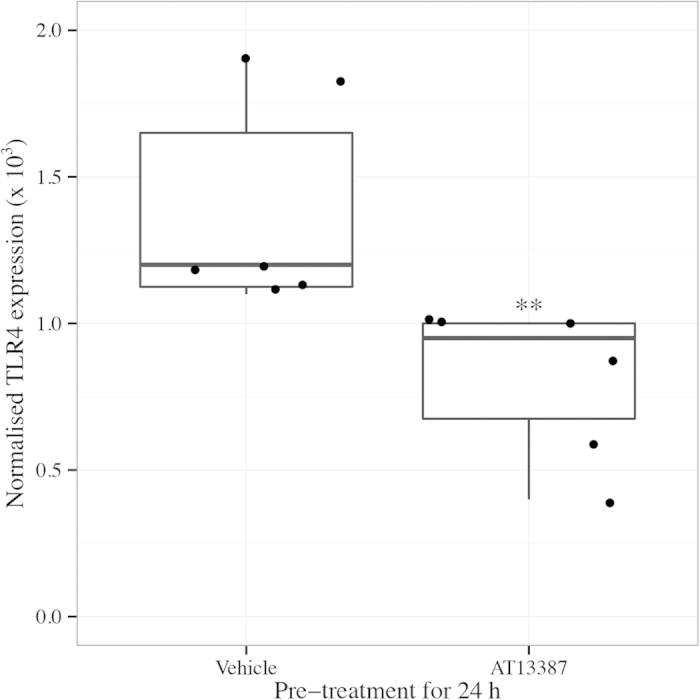
Renal TLR4 expression following AT13387 or vehicle pre-treatment and 24 h following renal IRI in FVB/n mice. FVB/n mice were pre-treated with AT13387 or 2HβC vehicle (n = 6 per group) and underwent renal IRI as per Fig. 1. The left kidney was harvested and stored in RNAlater for 24 h before being frozen. RNA was later extracted and converted to cDNA. PCR was then performed to determine TLR4 expression. TLR4 expression was normalised to GAPDH expression. Results are presented in a standard boxplot with individual results jittered. **p < 0.01 vs. 2HβC vehicle, Mann-Whitney U test.

**Figure 3:**
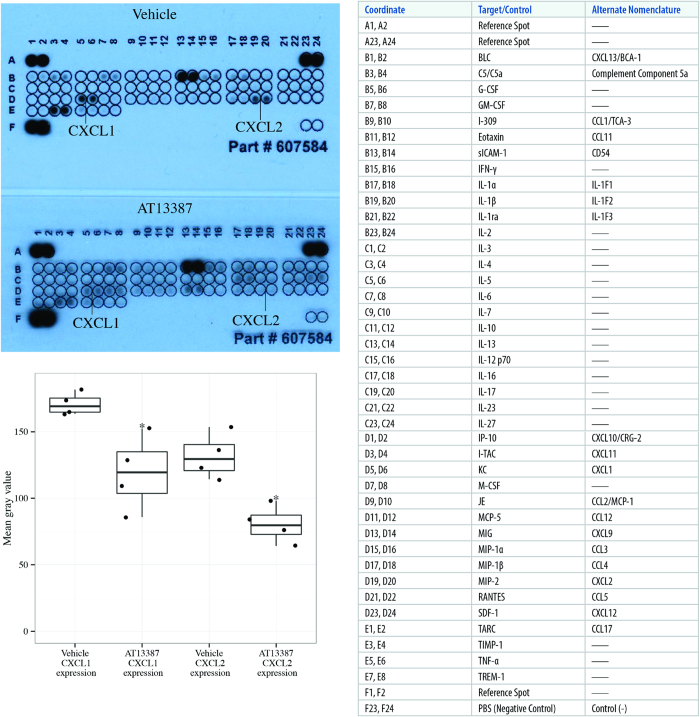
Renal cytokine expression following AT13387 or vehicle pre-treatment and 24 h following renal IRI in FVB/n mice. FVB/n mice were pre-treated with AT13387 or 2HβC vehicle (n = 4 per group) and underwent renal IRI as per Fig. 1. Following 24 h of recovery, the left kidney was harvested and snap frozen. Protein lysates were later prepared and an array panel was used to determine cytokine expression. The grid describes the cytokines assessed (right panel). A representative array is shown (upper left panel). Mean gray values were quantified using Image J and were used to reflect CXCL1 and CXCL2 expression. Results are presented in a standard boxplot with individual results jittered (lower left panel). *p < 0.05 vs. 2HβC vehicle, t-test.

On PCR analysis, in comparison to vehicle, AT13387 pre-treatment in FVB/n mice significantly reduced renal TLR4 expression 24 h following renal IRI (AT13387 vs. Vehicle, p < 0.01, Mann-Whitney U test) (Fig. 2). On cytokine array panel, in comparison to vehicle, AT13387 pre-treatment in FVB/n mice also reduced renal expression of chemokine (C-X-C motif) ligand 1 (CXCL1) and chemokine (C-X-C motif) ligand 2 (CXCL2) 24 h following renal IRI (AT13387 vs. 2HβC vehicle, p < 0.05, t-test) (Fig. 3). There were no other significant differences in cytokine or chemokine expression.

### Development of an *in vitro* model of sterile inflammatory signalling

#### Selection of a cell line

**Figure 4:**
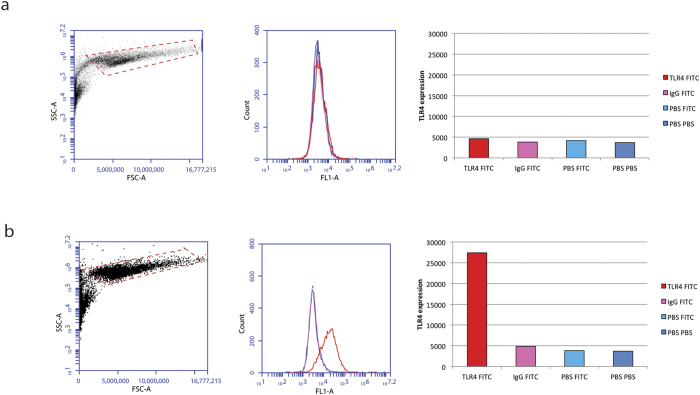
(**a**) TLR4 expression by HEK293 cells on flow cytometry (upper panel). Cells were trypsinised and collected at 50,000 cells per sample. After processing samples, results were acquired on a BD Accuri C6 flow cytometer. Cells were gated on the basis of forward (FSC-A) and side scatter (SSC-A). TLR4 expression (FLA-1) in HEK293 cells is represented by the red histograms, IgG-FITC negative control sample by the pink histograms, PBS-FITC negative control sample by the light blue histograms and PBS-PBS negative control sample by the dark blue histograms. (**b**) TLR4 expression by HEK293-TLR4 cells on flow cytometry (lower panel). Cells were analysed and results are reported as per Fig.4a.

The absence of TLR4 expression by HEK293 cells was confirmed by flow cytometry (Fig. 4a). Successful TLR4 transfection was validated in HEK293-TLR4 cells by flow cytometry (Fig. 4b).

#### Selection of a TR4-specific and sterile inflammatory ligand

**Figure 5:**
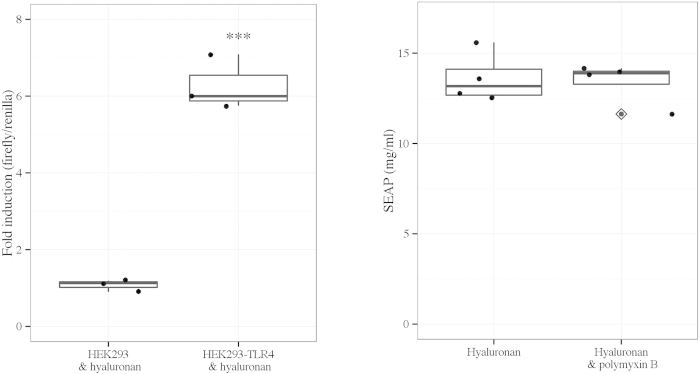
(**a**) Luciferase assay to determine NF-κB activity following NF-κB transfection and hyaluronan stimulation in HEK293 and HEK293-TLR4 cells (left panel). HEK293 and HEK293-TLR4 cells were divided at a cell density of 100,000 cells in 1 ml of growth medium per well of a 12-well plate. After 24 h, cells were transfected with 0.5 μg pGL4.32 [luc2P/NF-κB-RE/Hygro] plasmid DNA (experimental vector) using a ratio of DNA to Fugene HP of 3:1. A constitutively active control vector (pGL4.73 [hRluc/SV40]) was added at a ratio of experimental to control vector of 100:1. 24 h later, cells were treated with hyaluronan at a dose of 25 μg/ml. After 3 h incubation a luciferase assay was performed to determine firefly activity and renilla activity. Fold induction was calculated from the ratio of firefly to renilla and used to reflect NF-κB activity. Results are presented as 3 independent experiments in a standard boxplot with individual results jittered. The mean fold induction in HEK293 cells was 1.07 +/− 0.09 (standard error of the mean), i.e. there was no increase in NF-κB activity following hyaluronan stimulation. ***p < 0.001 vs. HEK293 & hyaluronan, t-test. (**b**) SEAP assay to determine NF-κB activity following hyaluronan stimulation with and without the presence of polymyxin B in HEK293-TLR4-NF-κB cells (right panel). HEK293-TLR4-NF-κB cells were divided at a cell density of 10,000 cells in 100 μl of growth medium per well of a 96-well plate. 24 h later, the medium was changed to medium containing 25 μg/ml of hyaluronan either with or without 50 μg/ml of polymyxin B. After 24 h incubation a SEAP assay was performed to determine NF-κB activity. Results are presented from 4 independent experiments in a standard boxplot with individual results jittered. Outlier data are highlighted by a dot with a diamond in the midline.

TLR4 was selectively expressed using TLR4-transfected cells (HEK293-TLR4). After ligand stimulation NF-κB activity was assessed by luciferase assay in comparison to TLR4-null cells (HEK293). Following hyaluronan stimulation there was increased NF-κB activity in HEK293-TLR4 cells, but an absence of NF-κB up-regulation in HEK293 cells (HEK293-TLR4 vs. HEK293, p < 0.001, t-test) (Fig. 5a). This indicates that hyaluronan is a TLR4-specific ligand in HEK293-TLR4 cells. The level of TLR4-mediated NF-κB activation assessed by SEAP assay following hyaluronan stimulation was not altered by the presence of polymyxin B, a drug that clears endotoxin contamination (Fig. 5b).

### Hsp90 inhibition represses sterile inflammatory signalling

#### Hsp90 inhibition abrogates TLR4-mediated NF-κB inflammatory activation

**Figure 6:**
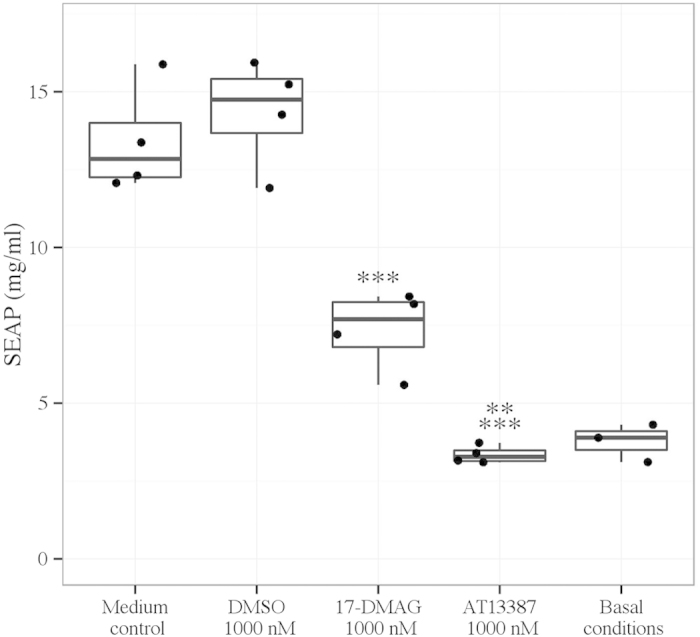
NF-κB activity determined by SEAP assay following pre-treatment with AT13387 or 17-DMAG and hyaluronan stimulation in HEK293-TLR4-NF-κB cells. Cells were divided as per Fig. 5b. 24 h later, cells were pre-treated with AT13387, 17-DMAG, DMSO vehicle or medium control for 6 h. With the exception of cells left in basal conditions, hyaluronan was added at a dose of 25 μg/ml for 24 h and a SEAP assay was performed to determine NF-κB activity. Results are presented from 3–4 independent experiments in a standard boxplot with individual results jittered. **p < 0.01 vs. 17-DMAG and ***p < 0.001 vs. DMSO vehicle, ANOVA.

In HEK293-TLR4-NF-κB cells, pre-treatment with AT13387 and 17-DMAG significantly reduced hyaluronan-mediated NF-κB activity assessed by SEAP assay (AT13387 1000 nM vs. DMSO vehicle, p < 0.001, 17-DMAG 1000 nM vs. DMSO vehicle, p < 0.001, ANOVA). In fact, pre-treatment with AT13387 reduced NF-κB activity assessed by SEAP assay following hyaluronan stimulation to a level equivalent of cells in basal conditions in normal culture medium. In addition, AT13387 was significantly more effective at reducing hyaluronan-mediated NF-κB activity assessed by SEAP assay than 17-DMAG (AT13387 1000 nM vs. 17-DMAG 1000 nM, p < 0.01, ANOVA) (Fig. 6).

#### Pre-treatment with AT13387 and 17-DMAG leads to breakdown of the IKK complex

**Figure 7:**
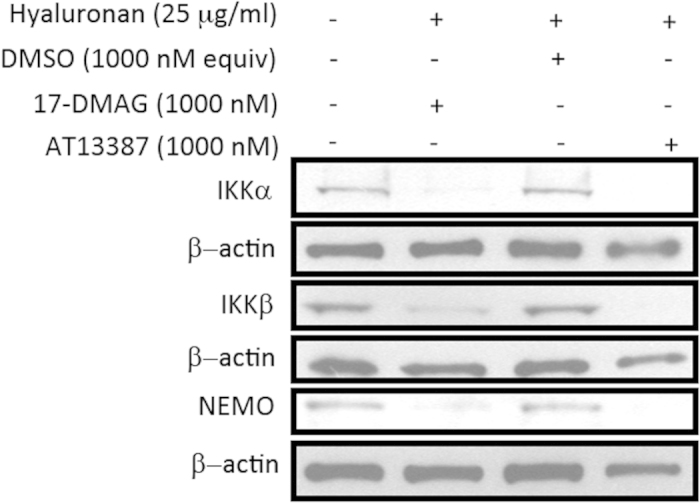
IKKα, IKKβ and NEMO levels on Western blotting following AT13387 or 17-DMAG pre-treatment and hyaluronan stimulation in HEK293-TLR4-NF-κB cells. HEK293-TLR4-NF-κB cells were divided at a cell density of 500,000 cells in 2 ml of growth medium per well of a 6-well plate. 24 h later, cells were then pre-treated with AT13387, 17-DMAG, DMSO vehicle or medium control for 12 h. With the exception of cells pre-treated with medium control, hyaluronan was added for 4 h. Whole-cell lysates were then prepared and analysed by Western blotting using antibodies to IKKα, IKKβ and NEMO, with β-actin being indicated as a loading control. Three different β-actin levels are presented because three separate Western blots were performed on the same samples to avoid antibody interactions. Cropped blots are displayed from each Western blot performed and show protein expression levels of IKKα, IKKβ and NEMO in the same samples. The gels were run under the same experimental conditions and original autoradiography is included in Supplementary Fig. 1.

Hsp90 inhibition, using pre-treatment with AT13387 and 17-DMAG led to breakdown of the IKK complex on Western blot following hyaluronan stimulation in HEK293-TLR4-NF-κB cells (Fig. 7 and original autoradiography in Supplementary Fig. 1).

## Discussion

In this study, AT13387 was tested in an established renal IRI model to assess efficacy of this agent in reducing renal IRI^10^. The experiments performed confirmed that AT13387 pre-treatment leads to both significant functional and morphological protection from renal IRI. This novel finding highlights AT13387 as a potentially exciting new therapy for reducing renal IRI.

Renal IRI predictably occurs in a range of clinical settings including surgery and vascular interventions. It can lead to an acute ischemic kidney injury, potentially life-threatening renal failure, as well as remote organ injury and multi-organ failure. Preventative treatment with AT13387 could be administered prior to anticipated ischemic insults, in individuals at high-risk of acute ischemic kidney injury (e.g. patients with pre-existing kidney disease, diabetes, or previous toxic drug and radiological contrast exposure).

Drugs designed to reduce renal IRI have an absolute requirement for low toxicity. Therefore, one of the main challenges going forward is to develop low toxicity drugs that can be safely utilised in humans. AT13387 is a novel small molecule Hsp90 inhibitor with a low toxicity profile in phase II human studies in oncology and therefore better translational potential than 17-DMAG in this context^8^. Furthermore, since there is patient safety data already available for AT13387, it may be possible to more rapidly translate this therapy into a clinical trial. However, prior to this an increased understanding of the mechanisms of protection offered by Hsp90 inhibition is required.

CXCL1 and CXCL2 are NF-κB target genes, and the expression of these pro-inflammatory chemokines is NF-κB dependent^14^,^15^. It has previously been identified that following renal IRI, there is increased expression of CXCL1 and CXCL2. Treatment with neutralizing antibodies to both CXCL1 and CXCL2 significantly improves kidney function within 48 hours of renal IRI^16^. The expression of CXCL1 and CXCL2 in the kidney was reduced following renal IRI in mice pre-treated with AT13387. There was also a significant reduction in renal TLR4 expression. This suggests an anti-inflammatory effect of AT13387. However, at just 24 hours following renal IRI, it is likely that protective effect of AT13387 may have involved programmed cell death rather than inflammation^17^. Therefore further mechanistic work was undertaken, and a carefully designed *in vitro* model was developed to simulate the sterile inflammatory environment of renal IRI.

HEK293 cells were selected specifically for these experiments since they transfect efficiently and have previously been reported to lack TLR4 expression^18^,^19^. This is in contrast to human renal tubular epithelial cells, which express basal levels of TLR4^20^.

HEK293 and HEK293-TLR4 cells were therefore considered ideal for identifying a suitable TLR4-specific ligand to explore *in vitro* the hypothesis that Hsp90 inhibition leads to repression of TLR4-mediated NF-κB inflammatory activation.

Hyaluronan was selected to stimulate the cells, as it is a damage associated molecular pattern molecule and a proposed endogenous TLR4 ligand that is released in increasing amounts during the sterile insult that comprises renal IRI^5^. Hyaluronan was characterised as a sterile TLR4-specific ligand in HEK293-TLR4 cells, and was therefore used to model sterile TLR4-specific NF-κB activation in further experiments. It was found that Hsp90 inhibition with AT13387 and 17-DMAG resulted in breakdown of IKKα, IKKβ and NEMO, which are the three subunits of the IKK complex^21^. This reduced highly specific TLR4-mediated NF-κB inflammatory activation by hyaluronan to the level of untreated cells left in basal conditions in normal culture medium.

Hsp90 inhibition has previously been used to experimentally treat TLR4-mediated autoimmune diseases^22^,^23^ and reduce tumour necrosis factor-alpha-mediated NF-κB activation^24^,^25^,^26^,^27^,^28^. However, this is the first study to identify that TLR4 signalling to NF-κB can be targeted by Hsp90 inhibitors. This finding therefore increases our current understanding of the potential role of Hsp90 in renal IRI, as well as inflammatory processes more generally.

In experimental renal IRI, transgenic mice that are missing TLR4^5^,^29^,^30^,^31^ or that have been treated with TLR4-blocking agents demonstrate a markedly protected phenotype^32^. Furthermore, de-activation of NF-κB with small interfering RNAs or decoy oligodeoxynucleotides targeting either IKKβ or NF-κB transcription factors, protects the kidney from IRI^33^,^34^,^35^.

The potential of Hsp90 inhibitors to interrupt TLR4-mediated NF-κB signalling is thus highly attractive from a translational perspective since it offers a pharmacological strategy to temporarily dampen NF-κB inflammatory activity. This is in contrast to ablative strategies targeting IKK that reduce NF-κB-mediated inflammation but also prevent subsequent NF-κB-mediated protection from apoptosis^36^.

Although numerous pathways can lead to NF-κB activation, IKK is a common point of convergence and activation of NF-κB occurs nearly universally via IKK-mediated degradation of IκB^6^. Indeed, it has previously been shown in HeLa cells that Hsp90 inhibition can prevent the formation of a proposed hetero-complex involving IKK and Cdc37/Hsp90, reducing the size of the complex and preventing activation of NF-κB^26^. It has also been observed in alveolar epithelial cells that treatment with Hsp90 inhibition induces dissociation of Hsp90 from the IKK complex, rendering the complex detergent insoluble and the NF-κB system impassive to cytokine stimulation^37^.

It therefore seems likely that Hsp90 inhibitors repress TLR4-mediated NF-κB activity primarily through IKK. The breakdown of all the major subunits of IKK by AT13387 and 17-DMAG following hyaluronan stimulation in this study would support this hypothesis. Although AT13387 was significantly more effective at reducing hyaluronan-mediated NF-κB activity, it does not appear to act through different mechanisms from 17-DMAG, However, as it is a smaller molecule Hsp90 inhibitor it may be more efficacious in this respect.

## Additional Information

**How to cite this article**: O’Neill, S. *et al.* Heat shock protein 90 inhibition abrogates TLR4-mediated NF-κB activity and reduces renal ischemia-reperfusion injury. *Sci. Rep.* **5**, 12958; doi: 10.1038/srep12958 (2015).

## Supplementary Material

Supplementary figure 1
