# Supplementary material for: Heat shock protein 90 inhibition abrogates TLR4-mediated NF-κB activity and reduces renal ischemia-reperfusion injury
Source: Sci Rep. 2015 Aug 7;5:12958. doi: 10.1038/srep12958 (PMC4528191; doi:10.1038/srep12958)
Supplement: Supplementary figure 1 [file srep12958-s1.pdf]

# Heat shock protein 90 inhibition abrogates TLR4-mediated NF-κB activity and reduces renal ischemia-reperfusion injury

**Short title**

Heat shock protein 90 inhibition reduces ischemic kidney injury


**Authors**

[1]Stephen O'Neill*, [1]Duncan Humphries, [1]George Tse, [1]Lorna P Marson, [1]Kevin Dhaliwal, [1]Jeremy Hughes, [2]James A Ross, [1]Stephen J Wigmore, [1]Ewen M Harrison

**Institution**

[1]MRC Centre for Inflammation Research, [2]MRC Centre for Regenerative Medicine, University of Edinburgh, Royal Infirmary of Edinburgh, 49 Little France Crescent, Edinburgh EH16 4SA

**Corresponding author***

Stephen O'Neill, MRC Centre for Inflammation Research, Tissue Injury and Repair Group, University of Edinburgh, Chancellor's Building, Royal Infirmary of Edinburgh, 49 Little France Crescent, Edinburgh EH16 4SA

Tel: 0044-7849592113

Fax: 0044-1312426520

E-mail: stephenoneill@doctors.org.uk

**Supplementary figure 1**

Scans of original autoradiography films of cropped Western blots displayed in figure 7 of the main manuscript

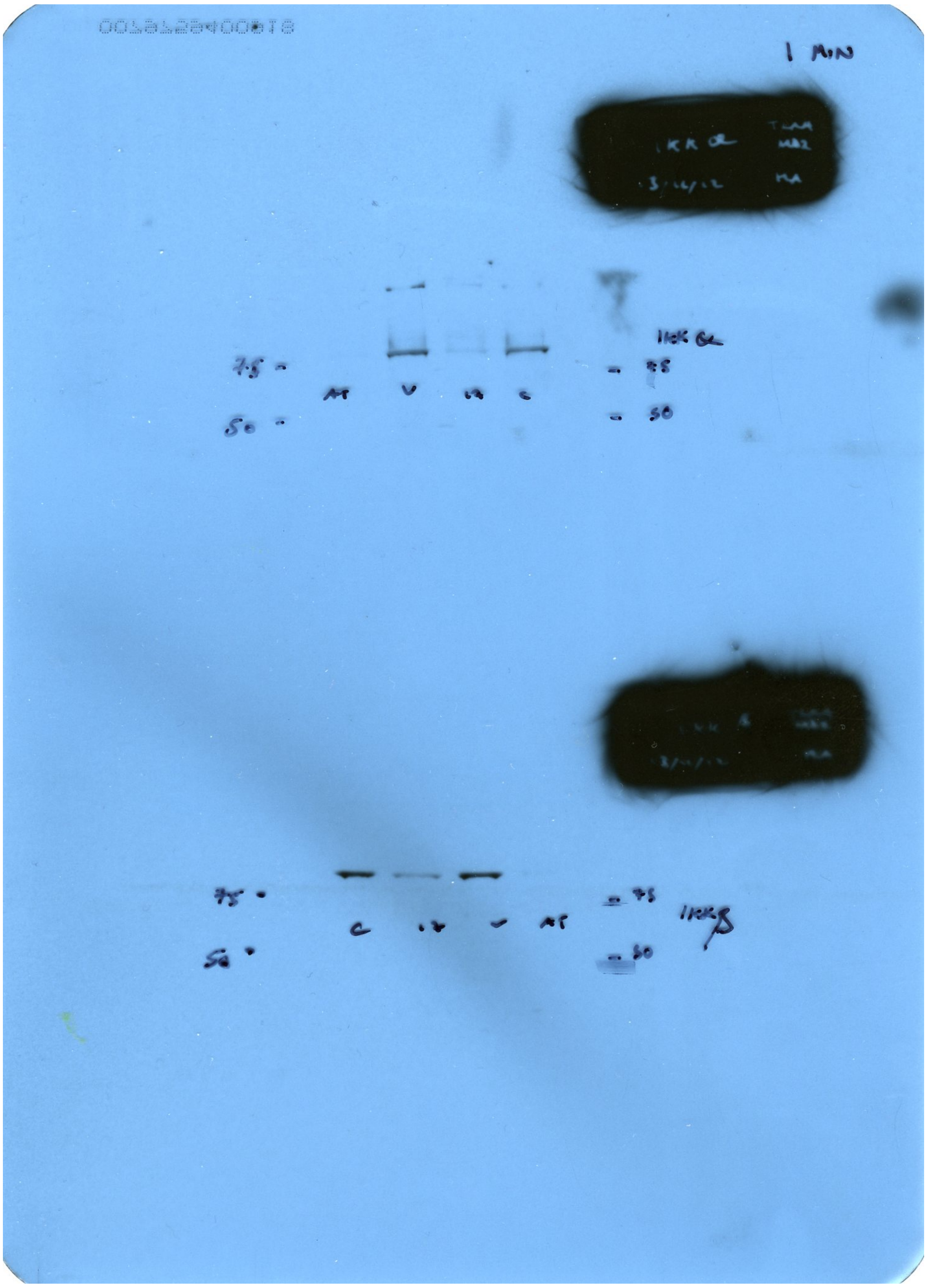


a) Western blots for IKKα (upper) and IKKβ (lower) are displayed. Annotation is as follows: C = control, V = Vehicle, 17 = 17-DMAG and AT = AT13387. Molecular weights for 75 kDa and 50 kDa have been marked. This original film was uploaded to a Gel Doc (Bio-Rad, Hemel, UK) using Quantity One software before being cropped for figure 7. Note in figure 7 the cropped blot of IKKα (upper) has been flipped horizontal so that the samples read left to right in the same order as the other cropped blots.

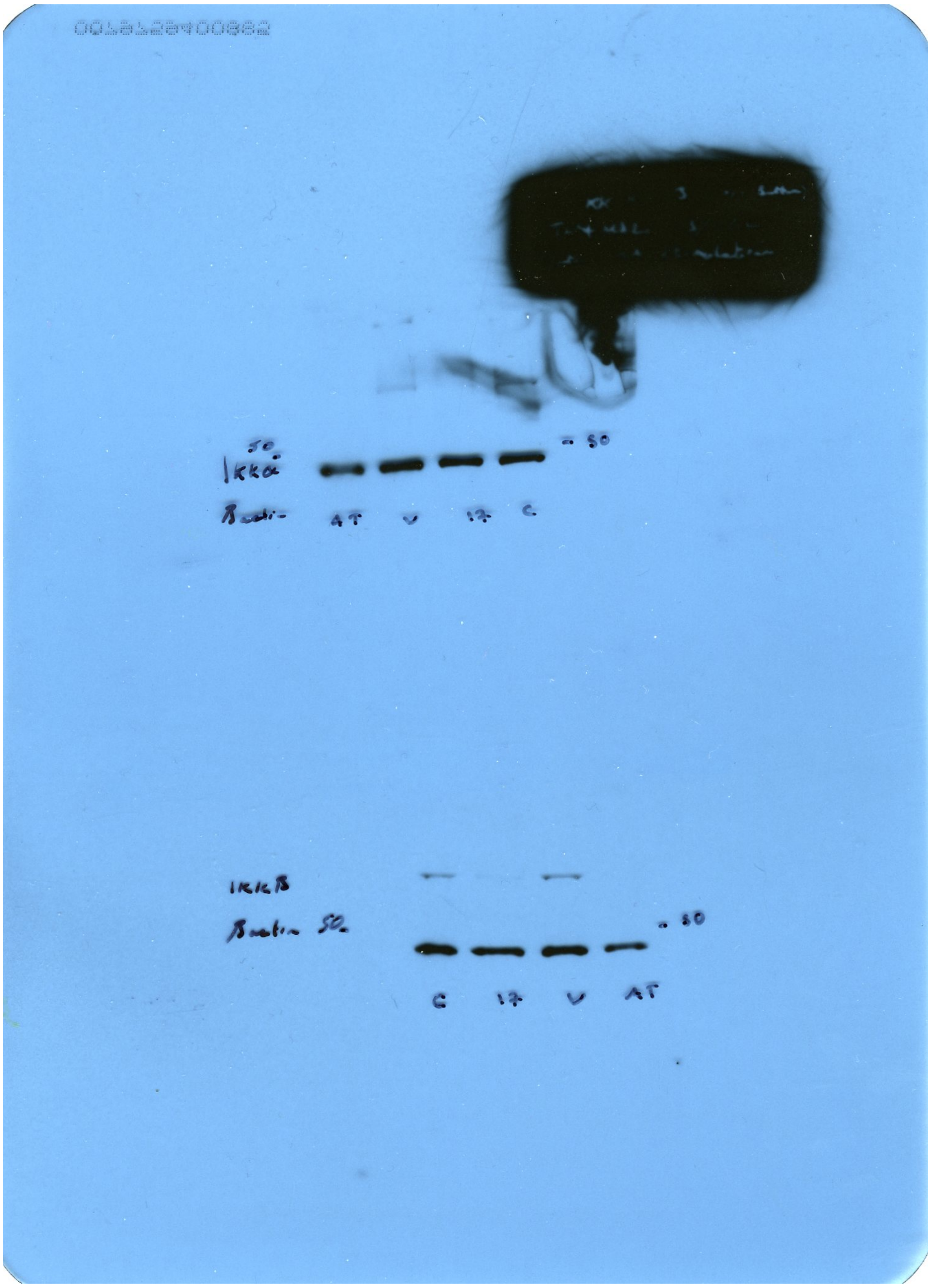


b) Re-probing β-actin in the blots performed for IKKα (upper) and IKKβ (lower). Annotation is as follows: C = control, V = Vehicle, 17 = 17-DMAG and AT = AT13387. The molecular weight for 50 kDa has been marked. In addition IKKα and IKKβ are still evident. This original film was uploaded to a Gel Doc (Bio-Rad, Hemel, UK) using Quantity One software before being cropped. Note in figure 7 the cropped blot of β-actin levels for the IKKα samples (upper) has been flipped horizontal so that the samples read left to right in the same order as the other cropped blots.

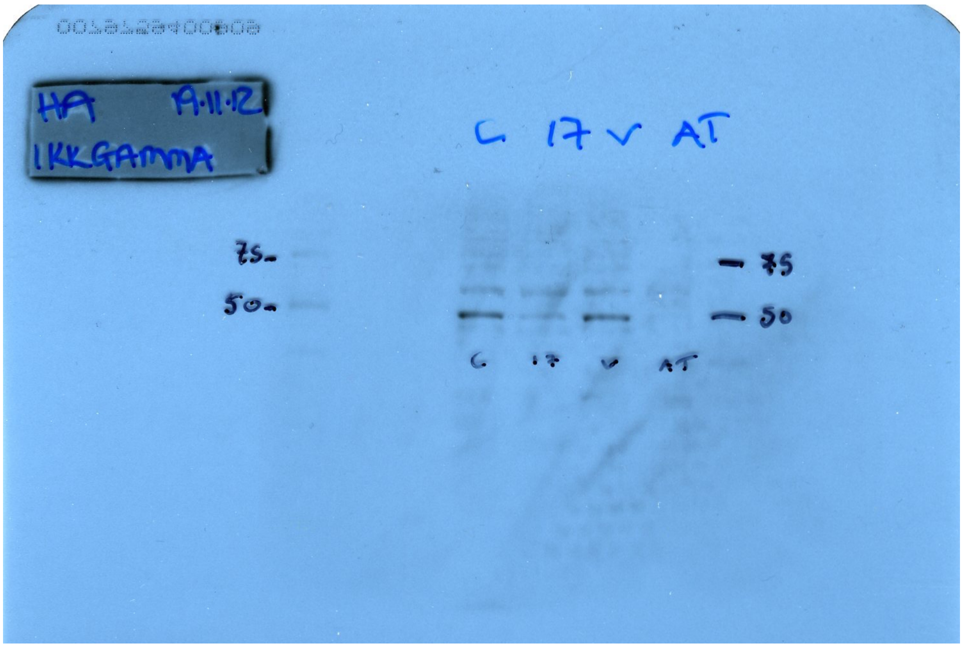


c) A Western blot for IKK Gamma (also known as NEMO) is displayed. Annotation is as follows: C = control, V = Vehicle, 17 = 17-DMAG and AT = AT13387. Molecular weights for 75 kDa and 50 kDa have been marked. This original film was uploaded to a Gel Doc (Bio-Rad, Hemel, UK) using Quantity One software before being cropped for figure 7.

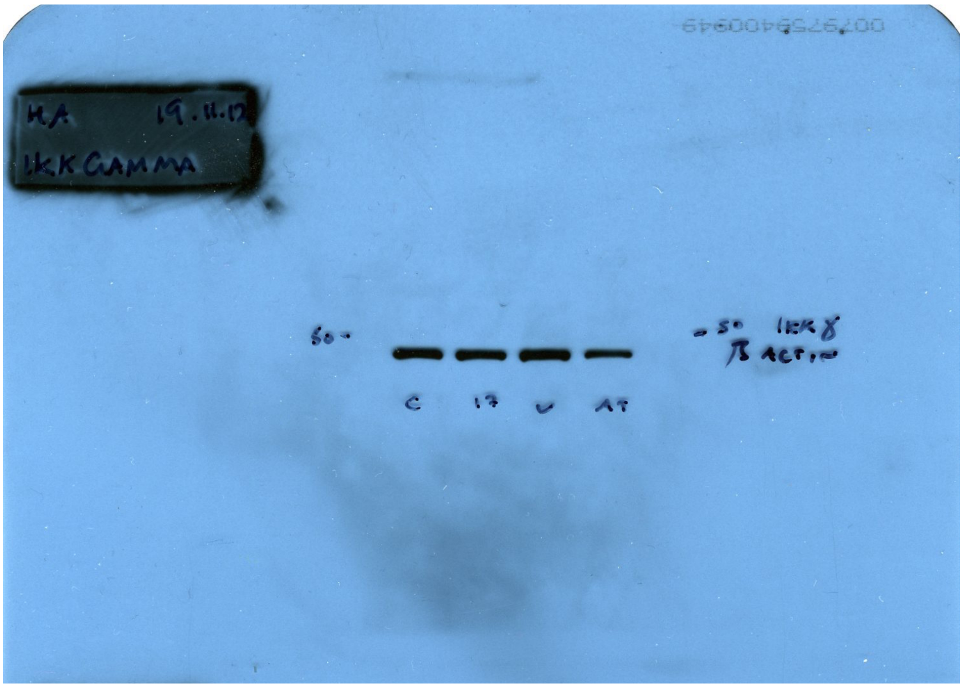


d) Re-probing β-actin in the blot performed for IKK Gamma (also known as NEMO). Annotation is as follows: C = control, V = Vehicle, 17 = 17-DMAG and AT = AT13387. The molecular weight for 50 kDa has been marked. This original film was uploaded to a Gel Doc (Bio-Rad, Hemel, UK) using Quantity One software before being cropped.
